# Optimal Dexmedetomidine Dose to Prevent Emergence Agitation Under Sevoflurane and Remifentanil Anesthesia During Pediatric Tonsillectomy and Adenoidectomy

**DOI:** 10.3389/fphar.2019.01091

**Published:** 2019-09-19

**Authors:** Yan-zhuo Zhang, Xue Wang, Jia-min Wu, Chun-yu Song, Xiao-guang Cui

**Affiliations:** Department of Anesthesiology, China and Heilongjiang Key Laboratory for Anesthesia and Critical Care, The Second Affiliated Hospital of Harbin Medical University, Harbin, China

**Keywords:** emergence agitation, dexmedetomidine, sevoflurane, remifentanil, tonsillectomy, adenoidectomy

## Abstract

**Background:** Emergence agitation (EA) is a common pediatric complication after sevoflurane anesthesia that can be prevented with dexmedetomidine. However, an inappropriate dose of dexmedetomidine can cause prolonged sedation and cardiovascular complications. Thus, we evaluated the optimal dose (ED95) of dexmedetomidine for preventing EA with sevoflurane and remifentanil anesthesia after pediatric tonsillectomy and adenoidectomy.

**Methods:** We enrolled American Society of Anesthesiologists (ASA) I and II children 3–7 years of age who underwent tonsillectomy with adenoidectomy. During induction, dexmedetomidine was infused for 10 min. Anesthesia was induced with sevoflurane and maintained with sevoflurane and remifentanil, resulting in a bispectral spectrum index (BIS) range from 40 to 60. Extubation time, surgical and anesthetic duration time, and duration time in the postanesthesia care unit (PACU) stay were recorded. EA [measured with Pediatric Anaesthesia Emergence Delirium (PAED) scores] and pain [measured with Face, Legs, Activity, Cry, Consolability (FLACC) scores] were assessed at extubation (E0), 15 min after extubation (E1), and 30 min after extubation (E2). If EA occurred, the next surgical procedure included increased dexmedetomidine by 0.1 μg/kg, and if not, the drug was reduced by 0.1 μg/kg.

**Results:** The 50% effective dose (ED50) of dexmedetomidine for preventing EA after sevoflurane and remifentanil anesthesia for tonsillectomy and adenoidectomy was 0.13 μg/kg, and its 95% confidence interval is 0.09–0.19 μg/kg; ED95 was 0.30 μg/kg, and its 95% confidence interval is 0.21–1.00 μg/kg.

**Conclusion:** Intravenous dexmedetomidine infusion at ED50 (0.13 μg/kg) or ED95 (0.30 μg/kg) during induction for 10 min can prevent half or almost all EA after sevoflurane and remifentanil anesthesia during pediatric tonsillectomy and adenoidectomy.

## Introduction

Emergence agitation (EA) is a common pediatric complication with sevoflurane anesthesia. EA incidence varies from 10% to 80% (Aono et al., 1997; Keaney et al., 2004; Vlajkovic and Sindjelic, 2007; Dahmani et al., 2010), and it is associated with complications, including increased heart rate, elevated blood pressure, dissatisfaction and anxiety, and fussy behaviors such as falling out of bed, removal of surgical dressings and intravenous catheters, and self-injury, which were generally associated with increased costs for additional medical care (Lerman et al., 1996; Welborn et al., 1996; Picard et al., 2000; Uezono et al., 2000). EA is driven by age, pain, surgery type, personality, rapid awakening, preoperative anxiety, and inhalation agents that were administered (Kuratani and Oi, 2008; Zhang et al., 2014). Many techniques and medications, which include regional block (Aouad et al., 2005; Ghosh et al., 2011; Slinha and Sood, 2012), premedication (Kim et al., 2013; Zhang et al., 2013), propofol (Picard et al., 2000; Uezono et al., 2000; Kim et al., 2012), µ-opioid agonists (Li et al., 2012; Liang et al., 2014), and α_2_-agonists (Boku et al., 2016; Lin et al., 2016), have been used to reduce pediatric EA.

Dexmedetomidine, a highly specific α_2_-adrenoceptor agonist with sedative, analgesic, and anxiolytic properties and few adverse effects, significantly reduces pediatric EA (Patel et al., 2010; Sato et al., 2010; Kim et al., 2014). Various doses (0.15–2.0 μg/kg) (Sun and Guo, 2014) of dexmedetomidine have been reported to prevent pediatric EA after sevoflurane anesthesia, but the optimal dose of dexmedetomidine is not known. Dexmedetomidine does not cause respiratory depression, but it does have cardiovascular effects (Kim et al., 2014), so use of the drug requires monitoring, especially for children (Buck, 2010). Thus, we sought to identify an optimal dose of dexmedetomidine to prevent EA without prolonged sedation and cardiovascular complications during tonsillectomy and adenoidectomy.

## Materials and Methods

### Study Oversight

Subjects underwent tonsillectomy and adenoidectomy at the Second Affiliated Hospital of Harbin Medical University at Harbin, Heilongjiang Province, China. This study was approved by the institutional ethics committee and registered at www.ClinicalTrials.gov (ChiCTR-OIh-17011790). Informed written consent to participate in this study was obtained from the parents and guardians of all of the children.

### Participants

Thirty-six children between 3 and 7 years of age with an American Society of Anesthesiologists (ASA) physical status I or II who were scheduled for tonsillectomy and adenoidectomy surgery were enrolled in this study. Subjects were selected using Dixon’s up-and-down sequential method (UDM) rather than random sequence. Patients with cardiac disease, developmental delay, abnormal upper airways, asthma, or a history of upper respiratory tract infection in the preceding 4 weeks were excluded. One child with cardiac disease, one child with developmental delay, one child with an abnormal upper airway, two children with asthma, and two children with a history of upper respiratory tract infection were excluded, and 29 children finished the experiments.

### Study Outcomes

We first determined the optimal dose of dexmedetomidine to prevent EA after pediatric tonsillectomy and adenoidectomy with sevoflurane and remifentanil anesthesia. We found the ED95 and ED50.

### Study Protocol

No premedication was given. Monitoring included electrocardiography (ECG), pulse oximetry (SpO_2_), noninvasive arterial blood pressure (NIBP), a depth of anesthesia monitor, and a BIS (VISTA monitoring system, Covidien, Ma). Anesthesia was induced with 3% sevoflurane with 5 L/min of oxygen, lidocaine (1 mg/kg), propofol (2–2.5 mg/kg), and atracurium (0.3 mg/kg). At the same time of induction, dexmedetomidine was infused (iv) for 10 min, and the starting dose for the first patient was 0.5 μg/kg. Anesthesia was maintained with 2–3% sevoflurane in approximately 50% oxygen with total inflow of 2 L/min and remifentanil (10 μg/kg/h, iv). Sevoflurane was adjusted to a BIS range of 40–60. Then, a 0.5% lidocaine and epinephrine mixture (1:200,000) in 3 ml were injected into the mucosa surrounding each tonsillar fossa for local anesthesia and vasoconstriction. Intravenous tramadol (2 mg/kg) and dexamethasone (0.1 mg/kg) were given after the induction of anesthesia for postoperative analgesia and to prevent postoperative nausea and vomiting. Sevoflurane was discontinued upon removal of medical mouth gag, and tracheal extubation was performed when patients began breathing spontaneously and coughing or had body movements. Then, children were transferred to the PACU, and EA was evaluated after extubation. If EA occurred, the next child’s dose of dexmedetomidine increased by 0.1 μg/kg compared with the previous one, and if not, it was lowered by 0.1 μg/kg. If severe EA occurred, the child was given propofol (0.5 mg/kg, iv). Postoperative pain was assessed with the Face, Legs, Activity, Cry, Consolability (FLACC) scale (Voepel-Lewis et al., 2010), and those with FLACC scores of 4 were given 0.5 μg/kg of fentanyl.

### Measurements

We recorded the duration of surgery and anesthesia, end-tidal sevoflurane concentration, extubation time, and PACU stay. Extubation time was measured from the cessation of sevoflurane to the point at which the patients’ eyes opened. EA severity was evaluated with the PAED (**Table 1**) scale devised by Sikich (Sikich and Lerman, 2004), and criteria appear in the table. EA incidence and severity were measured at extubation (E0), 15 min after extubation (E1), and 30 min after extubation (E2). Total PAED scores are sums for the five behaviors listed. EA was confirmed with an individual score of 4 points, a total PAED score that was no less than 10, or a comprehensive score that was greater than 15 (severe EA). Dexmedetomidine doses were determined using Dixon’s UDM (Dixon, 1991). Postoperative pain was assessed with FLACC at E0, E1, and E2. Rescue propofol and fentanyl were recorded if used. EA and postoperative pain were evaluated and recorded by a single anesthesiologist blinded to the sequence of inclusion of patients and the dose of dexmedetomidine administered to each patient.

**Table 1:** Pediatric Anesthesia Emergence Delirium (PAED) scale.

	Behavior frequency				

	None	Some	Modest	Much	Extreme
1. Child makes eye contact with caregiver.	4	3	2	1	0
2. Child’s action is purposeful.	4	3	2	1	0
3. Child is aware of his/her surroundings	4	3	2	1	0
4. Child is restless	0	1	2	3	4
5. Child is inconsolable	0	1	2	3	4

Items 1–3 are scored as follows: 4 = not at all, 3 = just a little, 2 = quite a bit, 1 = very much, and 0 = extremely. Items 4 and 5 are scored as follows: 0 = not at all, 1 = just a little, 2 = quite a bit, 3 = very much, and 4 = extremely.

### Statistics

Dixon’s UDM requires at least six failure–success pairs for statistical analysis; therefore, patient enrollment continued until six crossover pairs were observed. In our research, we observed nine crossover pairs. ED50 and ED95 of dexmedetomidine were estimated by calculating a modified isotonic estimator (Stylianou and Flournoy, 2002). The R 3.4.1 program was used for this calculation. The 95% confidence interval (CI) was obtained using a parametric bootstrap routine and calculated by a bias-corrected percentile method (Pace and Stylianou, 2007). Demographic and recovery data for children with EA and those without EA were compared with Fisher’s exact test and a Mann–Whitney *U* test. The PAED score and FLACC score for children with EA and those without EA were compared with repeated-measures ANOVA. Statistical analyses were performed with Statistical Product and Service Solutions (SPSS) 19.0 for Windows. Statistical significance was defined as *p* < 0.05.

## Results

Thirty-six children underwent tonsillectomy and adenoidectomy and were enrolled, and 29 children completed this study. Children with and without EA did not differ significantly with respect to age, weight, sex, surgery type or duration, extubation time, anesthetic duration, and PACU stay (*p* > 0.05). End-tidal sevoflurane concentration was recorded 15, 30, and 45 min after the administration of dexmedetomidine, and there was no significant difference in mean end-tidal sevoflurane concentration between the pediatric groups. There was no significant difference in mean FLACC scores between the groups. But PAED scores in the EA group are significantly higher than those in the without-EA group (*p* < 0.05) (**Table 2**).

**Table 2:** Demographic and surgical characteristics of children with and without EA (mean ± SD).

	With EA (*N* = 12)	Without EA (*N* = 17)	*P*
Gender (female/*n*)	4/12	8/17	0.70
Weight (kg)	24.09 ± 6.49	21.32 ± 3.26	0.12
Age (year)	5.45 ± 1.29	5.29 ± 1.10	0.45
Tonsillectomy/adenotonsillectomy	11/12	15/17	1.00
Duration of operation (min)	31.00 ± 6.81	31.82 ± 14.43	0.85
Duration of anesthesia (min)	55.29 ± 7.45	53.64 ± 12.31	0.52
Extubation time (min)	13.88 ± 4.09	16.95 ± 7.57	0.09
End-tidal sevoflurane concentration (%)	3.18 ± 0.81	3.21 ± 1.03	0.84
PACU stay time (min)	6.82 ± 2.52	6.06 ± 2.01	0.72
FLACC	3.44 ± 0.12	3.18 ± 0.10	0.11
PAED	7.69 ± 0.44	12.03 ± 0.52	0.00

A p <0.05 signifies significant differences between groups. EA, emergence agitation; PACU, postanesthesia care unit; FLACC, Face, Legs, Activity, Cry, Consolability; PAED, Pediatric Anaesthesia Emergence Delirium.

**Figure 1:**
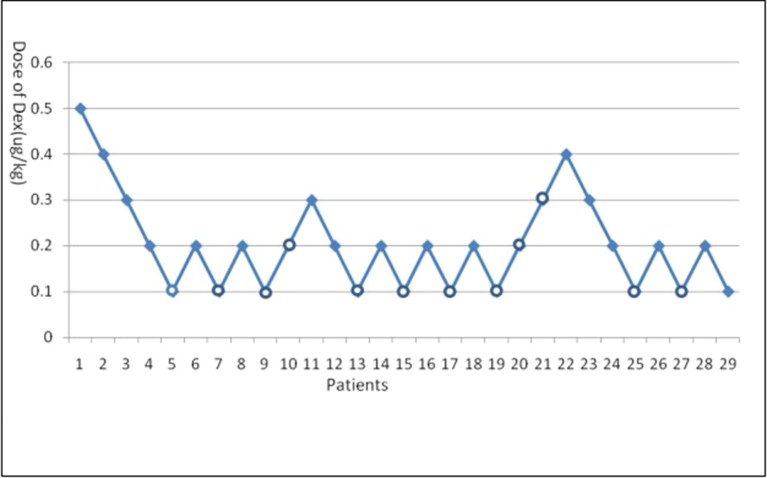
Emergence agitation (EA) using Dixon’s up-and-down method. Crossover pairs from success (solid square) to failure (hollow circle).

Dexmedetomidine dose success/failure data appear in **Figure 1**. From this figure, the ED95 and ED50 of dexmedetomidine to prevent EA after sevoflurane and remifentanil anesthesia are 0.30 μg/kg (its 95% CI is 0.21–1.00 μg/kg) and 0.13 μg/kg (its 95% CI is 0.09–0.19 μg/kg), respectively.

## Discussion

Dexmedetomidine is a selective α_2_-agonist that has a short half-life. Because of its sedative, analgesic, and anxiolytic properties, it is used to prevent pediatric EA during tonsillectomy and adenoidectomy. A single dose or continuous infusion of dexmedetomidine can reduce pediatric EA after sevoflurane anesthesia (Zhang et al., 2014; Makkar et al., 2016; TBedirli et al., 2017), but an optimal dose has not yet been determined. We report the ED95 and ED50 for dexmedetomidine (0.30 and 0.13 μg/kg, respectively), determined using Dixon’s UDM, and this dose offers less EA and less risk of complications related to dexmedetomidine overdose.

Dixon’s UDM was designed to estimate the median threshold, including the ED50 or EC50 (Dixon, 1991; Oron, 2006; Pace and Stylianou, 2007), it has many advantages such as small sample size, simple operation, and relatively accurate results. And it can estimate the ED90/EC90 or ED95/EC95 by extrapolation in a UDM study (Lee et al., 2017). Kim’s group reported that a dexmedetomidine bolus (0.38 μg/kg) could prevent 95% of 2- to 12-year-old pediatric EA cases after tonsillectomy and adenoidectomy with desflurane anesthesia (Kim et al., 2015). Considering the greater incidence of EA after sevoflurane anesthesia and that EA in with those aged 3–7 years is severe than that in others (Aono et al., 1997; Keaney et al., 2004; Vlajkovic and Sindjelic, 2007; Dahmani et al., 2010; Choi et al., 2015), we selected 0.5 μg/kg of dexmedetomidine as the initial dose and observed no adverse reactions, such as bradycardia, hypotension, or prolonged recovery time (Mason et al., 2009). Atropine (0.02 mg/kg) as antisialagogue at the time of induction antagonized potential bradycardia induced by dexmedetomidine, which has a half-life of 2 h and is rapidly metabolized. In Kim’s study (Kim et al., 2015), they selected a dosing increment of dexmedetomidine of 0.1 µg/kg. We increased and decreased the increment in our preliminary experiment, but it is inappropriate, so we also selected 0.1 µg/kg as an increment dose of dexmedetomidine.

Postoperative pain is a cause of agitation (Lynch et al., 1998); but even with analgesia (Seo et al., 2011), postanesthetic EA has been observed (Weldon et al., 2004), or there was an absence of pain (Cravero et al., 2000). To address this, we gave tramadol 2 mg/kg (iv) during the induction of anesthesia to minimize pain effects. During surgery, local anesthesia with 0.5% lidocaine and epinephrine (1:200,000) was used to reduce postoperative pain. There were no differences in FLACC scores between the two groups.

During pediatric anesthesia, sevoflurane is often paired with ketamine, propofol, dexmedetomidine, clonidine, midazolam, fentanyl, remifentanil, or sufentanil to prevent EA (Wang et al., 2017). Remifentanil is an appropriate synthetic short-acting opioid commonly combined with volatile anesthesia for pediatric patients. Remifentanil prevents EA after sevoflurane anesthesia as well (Dong et al., 2010; Na et al., 2013; Wang et al., 2017). Since we still observed EA, we used prophylactic dexmedetomidine to prevent EA and noted the ED50 and ED95 values. The study is limited by a small sample size although statistical power was sufficient. Although the *p* value of body weight between two groups is 0.12, the mean body weight in the EA group is slightly bigger than that in the without-EA group. This may have a slight impact on the final ED50/ED95 outcome. More research is required to confirm our preliminary data and our dexmedetomidine dose. And our dexmedetomidine dose must be verified with other preoperative medications, so that it can be better used in clinical practice.

## Conclusion

Intravenous dexmedetomidine infusion at ED50 (0.13 μg/kg) or ED95 (0.30 μg/kg) during induction for 10 min can prevent half or almost all EA after sevoflurane and remifentanil anesthesia during pediatric tonsillectomy and adenoidectomy.

## Data Availability

All datasets generated for this study are included in the manuscript/supplementary files.

## Ethics Statement

The studies involving human participants were reviewed and approved by the institutional ethics committee of Harbin Medical University and registered at www.ClinicalTrials.gov (the ethic number is ChiCTR-OIh-17011790). Written informed consent to participate in this study was provided by the participants’ legal guardian/next of kin. Written informed consent was obtained from the individual(s), and minor(s)’ legal guardian/next of kin, for the publication of any potentially identifiable images or data included in this article.

## Author Contributions

All of the authors had access to the data and guarantee the integrity and accuracy of the data and analyses. The first author wrote the first draft of the manuscript, and all of the authors participated in the subsequent drafts. All of the authors agreed on submitting the paper, and there was no commercial support for the study.

## Conflict of Interest Statement

The authors declare that the research was conducted in the absence of any commercial or financial relationships that could be construed as a potential conflict of interest.
